# Non-steroidal anti-inflammatory drugs and clinical outcomes in patients with COVID-19

**DOI:** 10.3389/fcimb.2022.935280

**Published:** 2022-10-17

**Authors:** Jing Zhang, Hongguang Sheng, Xiaoyi Tang, Panpan Xia, Zhangwang Li, Minxuan Xu, Jianyong Ma, Yunfeng Shen, Peng Yu, Xiao Liu

**Affiliations:** ^1^ Department of Anesthesiology, The Second Affiliated Hospital of Nanchang University, Nanchang, China; ^2^ Department of Metabolism and Endocrinology, The Second Affiliated Hospital of Nanchang University, Nanchang, China; ^3^ Institute for the Study of Endocrinology and Metabolism in Jiangxi Province, Nanchang, China; ^4^ The Second Clinical Medical College of Nanchang University, Nanchang, China; ^5^ Department of Pharmacology and Systems Physiology, University of Cincinnati College of Medicine, Cincinnati, OH, United States

**Keywords:** nonsteroidal anti-inflammatory drugs, COVID-19, mortality, discharge, severity

## Abstract

The use of non-steroidal anti-inflammatory drugs (NSAIDs) in patients with coronavirus disease 2019 (COVID-19) has raised great concerns. The effect of NSAIDs on the clinical status of COVID-19 remains in question. Therefore, we performed a *post-hoc* analysis from the ORCHID trial. Patients with COVID-19 from the ORCHID trial were categorized into two groups according to NSAID use. The 28-day mortality, hospitalized discharge, and safety outcomes with NSAIDs for patients with COVID-19 were analyzed. A total of 476 hospitalized patients with COVID-19 were included; 412 patients (86.5%) did not receive NSAIDs, while 64 patients (13.5%) took NSAIDs as regular home medication. Patients who took NSAIDs did not have a significant increase in the risk of 28-day mortality (fully adjusted: hazard ratio [*HR*]: 1.12, 95% *CI*: 0.52–2.42) in the Cox multivariate analysis. Moreover, NSAIDs did not decrease hospital discharge through 28 days (fully adjusted: *HR*: 1.02, 95% *CI*: 0.75–1.37). The results of a meta-analysis including 14 studies involving 48,788 patients with COVID-19 showed that the use of NSAIDs had a survival benefit (summary risk ratio [*RR*]: 0.70, 95% *CI*: 0.54–0.91) and decreased the risk of severe COVID-19 (summary: *RR*: 0.79, 95% *CI*: 0.71–0.88). In conclusion, the use of NSAIDs is not associated with worse clinical outcomes, including 28-day mortality or hospital discharge in American adult hospitalized patients with COVID-19. Based on current evidence, the use of NSAIDs is safe and should not be cautioned against during the COVID-19 pandemic. Ongoing trials should further assess in-hospital treatment with NSAIDs for patients with COVID-19.

## Introduction

Coronavirus disease 2019 (COVID-19), an acute respiratory disease caused by the novel severe acute respiratory syndrome coronavirus 2 (SARS-CoV-2), is currently prevalent worldwide (Barillà et al., 2020; Coto et al., 2021). Non-steroidal anti-inflammatory drugs (NSAIDs) include non-selective cyclooxygenase (COX) inhibitors (e.g., aspirin, ibuprofen, and naproxen), as well as selective COX2 inhibitors (e.g., celecoxib, rofecoxib, and etoricoxib). NSAIDs are inexpensive and readily available drugs widely applied for pain relief and fever reduction (Bacchi et al., 2012). Hence, the use of NSAIDs (e.g., ibuprofen and acetaminophen) is very common for symptomatic treatment in patients with COVID-19 (Vaja et al., 2021). Recently, NSAIDs were suggested to worsen the clinical status of patients with COVID-19 through the upregulation of angiotensin-converting enzyme 2 (ACE2) receptors. Case reports and several observational studies found that ibuprofen might aggravate the outcomes following NSAID use; however, this finding was not replicated in several subsequent reports. To date, no consensus guidelines are available regarding aspirin use in COVID-19 due to a lack of evidence. The Health Minister of France recommended that paracetamol (acetaminophen) be used as a first-line antipyretic agent over NSAIDs. A recent national retrospective cohort from South Korea also suggested that the use of NSAIDs increased the risk of ischemic stroke in patients with COVID-19, prompting safety concerns regarding their use among this population (Jeong et al., 2021). In contrast, a prospective cohort showed ibuprofen and other NSAIDs are not significantly associated with mortality or the risk of hospital admission (Abu Esba et al., 2021). Other administrations (such as the US Food and Drug Administration and the World Health Organization) stated that NSAIDs should not be changed in current clinical practice until further evidence becomes available (Day, 2020).

In light of the NSAID–COVID-19 debate and the rapidly unfolding situation regarding the current pandemic, we performed a post-hoc analysis of the Outcomes Related to COVID-19 Treated with Hydroxychloroquine Among Inpatients with Symptomatic Disease (ORCHID) trial, which is a blinded, placebo-controlled randomized trial conducted across 34 hospitals in the United States. We aimed to 1) investigate the associations of NSAID use with clinical outcomes, 2) assess the safety (particularly in terms of vascular complications) of NSAIDs in patients with COVID-19, and 3) conduct a meta-analysis to comprehensively evaluate the effect of NSAIDs on patients with COVID-19.

## Materials and methods

### Data source

We obtained data from ORCHID (Casey et al., 2020). Briefly, ORCHID is a multicenter, blinded, randomized clinical trial (RCT) that compared hydroxychloroquine with a placebo on the clinical status of hospitalized patients with COVID-19. The trial included 479 hospitalized patients with COVID-19 in 43 US hospitals between 2 April 2020 and 19 June 2020. The inclusion criteria were as follows: 1) adults hospitalized with COVID-19 < 48 h with polymerase chain reaction-confirmed positive SARS-CoV-2 and 2) symptoms of respiratory illness for less than 10 days. The main exclusion criteria were treatment with hydroxychloroquine or chloroquine or medications that prolong the QTC interval to >500 ms within 10 days of hospitalization. The primary outcome was clinical status 14 days after randomization as assessed with the 7-category ordinal scale (the COVID Outcomes Scale) recommended by the World Health Organization. The second outcome was the COVID Outcomes Scale and the clinical outcomes (including 14- and 28-day mortality, an extracorporeal membrane oxygenation (ECMO) event, or intensive care unit (ICU) admission). Patients were followed up for death until 28 days following hydroxychloroquine randomization using in-hospital records and telephone follow-up after discharge. The Prevention and Early Treatment of Acute Lung Injury (PETAL) Clinical Trials Network Clinical Coordinating Center reviewed all the information to ensure data quality. A central institutional review board at Vanderbilt University Medical Center approved the ORCHID. Informed consent for participation was obtained from the patients or legally authorized representatives. The main results of the ORCHID have been published (Self et al., 2020). Notably, the investigators of the RCTs were not involved in this study. Two patients were lost to follow-up over the 28 days, and one had missing baseline characteristics. Finally, 476 patients were included in this study. Reporting of the research conforms to the Strengthening the Reporting of Observational Studies in Epidemiology (STROBE) statement (Von Elm et al., 2007).

### Exposure

NSAID users were defined as those regularly taking NSAIDs as home medication according to the electronic health record. Non-users were defined as those who did not regularly take NSAIDs as a home medication.

### Outcomes

The outcomes were all-cause mortality through 28 days after trial entry and discharge through 28 days after hospital admission. The detailed definitions of these outcomes can be found in previous descriptions.

### Covariates

Potential confounders at baseline considered to be associated with NSAID use and clinical outcomes were collected, including demographics (age, sex, and race), comorbidities (body mass index, hypertension, diabetes, chronic kidney disease, coronary artery disease, and chronic obstructive pulmonary disease), laboratory measurements (white blood cell count, platelet count, creatinine, aspartate aminotransferase, and alanine aminotransferase), duration of symptoms at baseline, total Sequential Organ Failure Assessment (SOFA) score at enrollment, symptoms of acute respiratory infection (shortness of breath, cough, and fever), chronic medication history (angiotensin-converting enzyme inhibitor and angiotensin II receptor blocker, and corticosteroids), and inpatient treatments (e.g., corticosteroid, tocilizumab, and azithromycin).

### Statistical analysis

Continuous variables are expressed as the means with standard deviations if they conformed to a normal distribution or medians with interquartile ranges (IQRs) if they did not. The differences between groups for continuous variables were compared using the unpaired Student’s t-test (normal distribution) or Wilcoxon–Mann–Whitney tests (non-normal distribution). Categorical variables, reported as counts and percentages, were compared between groups using the χ^2^ test. For non-normally distributed categorical variables, the Kruskal–Wallis test was used. Survival analysis was performed using Kaplan–Meier (K-M) estimates tested by the log-rank method. Cox proportional hazards models were used to calculate the adjusted risk estimates (i.e., hazard ratios [*HR*s] and their confidence intervals [*CI*s]). The selection of adjusted covariates in the multivariable models was based on the backward stepwise method with a significance level of <0.10, including all the baseline factors. Safety was analyzed with simple logistic regression to calculate the crude *OR*. Meta-analysis is conducted as a supplemental analysis to systematically assess the impact of NSAIDs and prognosis in patients with COVID-19 by searching PubMed and EMBASE up to February 2021. All statistical analyses were performed using SPSS Statistics version 26.0 (IBM) and R (version 4.0.1) software. A two-sided *p*-value of less than 0.05 was considered statistically significant.

### Supplementary analyses

To evaluate the robustness of the findings, we conducted a sensitivity analysis by 1) extending the definition of 28-day mortality to in-hospital mortality and 2) assessing ICU admission as the second outcome by excluding the population not admitted to the ICU at baseline.

## Results

### Baseline characteristics

We included 476 hospitalized patients with COVID-19 (mean age, 57 [IQR 44-68] years; female ratio, 44.2%). The baseline characteristics of all patients with COVID-19 categorized as NSAID users are shown in **Table 1**. Overall, NSAID users were common (13.3%), with 412 non-users and 64 NSAID users. Compared with non-users, NSAID users were older, more likely to use corticosteroids at home, and more likely to have chronic obstructive pulmonary disease (COPD). There was no significant difference in the in-hospital medication (e.g., azithromycin) administered or the proportion of acute respiratory infection symptoms, chest imaging, or the total SOFA score between NSAID users and non-users.

**Table 1:** Baseline characteristics of included hospitalized COVID-19 patients stratified by NASIDs user

	Non-NSAID users (n = 412)	NSAID users (n = 64)	*p*
**Demography**			
Age, years	56.0 (16.5)	62.1 (15.6)	0.006
Sex, male (%)	231 (56.1)	35 (55.6)	0.84
BMI, kg/m2	32.7 (9.9)	33.8 (8.8)	0.38
Ethnic, Hispanic, or Latino (%)	154 (38.6)	22 (34.4)	0.61
**Chronic health conditions**			
Peripheral vascular disease (%)	15 (3.6)	4 (6.2)	0.52
COPD (%)	29 (7.0)	10 (15.6)	0.04
Hypertension (%)	210 (51.0)	41 (64.1)	0.07
Coronary artery disease (%)	33 (8.0)	9 (14.1)	0.18
Diabetes mellitus (%)	143 (34.7)	21 (32.8)	0.88
Moderate to severe kidney disease (%)*	34 (8.3)	8 (12.5)	0.38
**Home medication**			
Corticosteroids (%)	34 (8.3)	13 (20.3)	0.005
ACE inhibitors (%)	63 (15.3)	12 (18.8)	0.60
ARB (%)	34 (8.3)	7 (10.9)	0.64
**Symptoms of acute respiratory infection**			
Cough (%)	243 (59.0)	38 (59.4)	1.00
Fever (%)	241 (58.5)	29 (45.3)	0.07
Shortness of breath (%)	290 (70.4)	50 (78.1)	0.26
Sore throat (%)	29 (7.0)	5 (7.8)	1.00
Total SOFA score	3.07 (2.74)	3.59 (3.27)	0.17
**Measurements**			
Systolic blood pressure, mmHg	111 [100, 124]	112 [100, 123]	0.51
White blood cell count, /mm3	6,000 [4,292, 7,900]	5,310 [3,900, 7,300]	0.32
Hemoglobin, g/dL	12.9 [11.5, 14.2]	12.45 [10.6, 14.2]	0.19
Sodium, mmol/L	136.0 [134.0, 138.0]	136.0 [133.0, 139.0]	0.96
Potassium, mmol/L	3.9 [3.6, 4.2]	3.8 [3.5, 4.3]	0.36
BUN, mg/dL	14.0 [10.3, 24.0]	19.5 [12.0, 37.5]	0.007
Troponin, ng/mL	0.01 [0, 0.1]	0.01 [0, 0.1]	0.81
AST, U/L	41.0 [29.8, 64.3]	44.5 [28.0, 66.8]	0.74
ALT, U/L	31.0 [20.0, 54.0]	33.0 [20.0, 62.0]	0.55
ALP, U/L	75.0 [58.0, 94.0]	82.0 [61.0, 100.0]	0.67
**Bilateral opacities/infiltrates (%)**	249 (63.2)	41 (65.1)	0.88
**Pre-medication up to randomization**			
Hydroxychloroquine (%)	4 (1.0)	0 (0.0)	0.96
Remdesivir (%)	21 (5.1)	3 (4.7)	0.90
Lopinavir/ritonavir (%)	0 (0)	0 (100.0)	NA
Corticosteroids (%)	29 (7.0)	6 (9.4)	0.68
Tocilizumab (%)	5 (1.2)	0 (0.0)	0.82
Sarilumab (%)	0 (0)	0 (0)	NA
Interferon (%)	0 (0.0)	1 (1.6)	0.28
Azithromycin (%)	124 (30.1)	24 (37.5)	0.30
**Medication between randomization and hospital discharge**			
Corticosteroids (%)	72 (17.5)	15 (23.4)	0.33
Tocilizumab (%)	23 (5.6)	4 (6.2)	1.00
Sarilumab (%)	0 (0)	0 (0)	NA
Interferon (%)	0 (0)	0 (0)	NA
Immunomodulating medication (%)	3 (0.7)	1 (1.6)	1.00
Randomization to hydroxychloroquine (%)	205 (49.8)	32 (50.0)	1.00

Mean (interquartile range) for nonnormally distributed data, Mean ± (standard deviation) for normally distributed data, and n (%) for categoric variables.BMI, body mass index; COPD, chronic obstructive pulmonary disease; ACEi, angiotensin-converting enzyme inhibitors; ARB, Angiotensin Receptor Blockers; SOFA, Sequential Organ Failure Assessment; BUN, blood urea nitrogen; ALT, alanine aminotransferase; AST, aspartate aminotransferase; ALP, alkaline phosphatase; NSAIDs, Non-steroidal anti-inflammatory drugs. *Moderate to severe kidney disease was defined as Cr >3, ESRD, chart diagnosis of CKD stage 5 (eGFR <15 mL/min/1.73m^2^) not on dialysis.

### Outcomes

**Figure 1:**
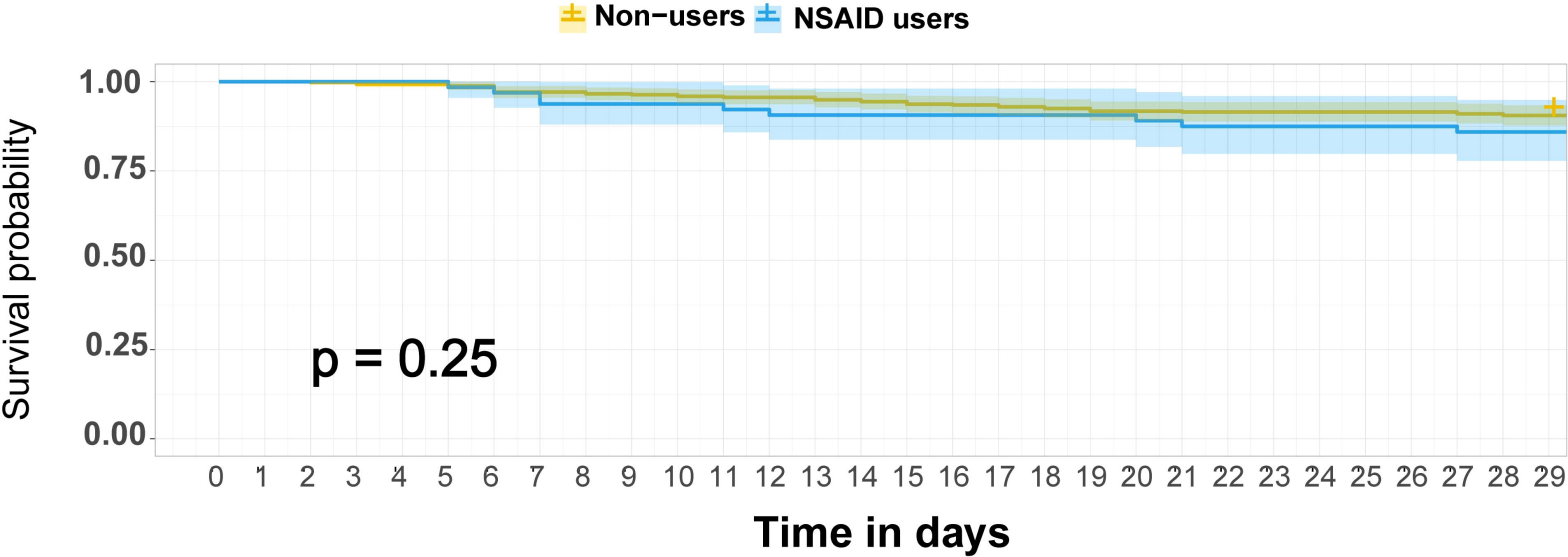
K-M survival curves for 28-day mortality among NSAID users and non-users among hospitalized patients with COVID-19. The survival curves are survival function (Kaplan–Meier) curves with a *p*-value calculated by the log-rank test. Patients were followed up for death until 28 days after randomization in ORCHID using in-hospital records and telephone follow-up. COVID-19, coronavirus disease 2019; ORCHID, Outcomes Related to COVID-19 Treated With Hydroxychloroquine Among Inpatients With Symptomatic Disease; K-M, Kaplan–Meier; NSAID, non-steroidal anti-inflammatory drug.

There were 45 patients who died within 28 days of trial inclusion, 39 of whom were non-users and 9 who were NSAID users. As shown in **Figure 1**, the K-M curves showed no significant difference in 28-day mortality between non-users and NSAID users (*p* = 0.48). The results of the univariable and multivariable Cox regression analyses are shown in **Table 2**. NSAIDs showed no significantly decreased survival benefit in hospitalized patients with COVID-19, either in the univariable analysis or after adjustment for all confounders (fully adjusted: *HR*: 1.12, 95% *CI*: 0.52–2.42).

**Table 2:** Association between use of NSAIDs, 28-day mortality, and hospital discharge in patients with COVID-19.

Outcomes	Non-users Cases/N	NSAID users Cases/N	Crude	Model 1^#^	Model 2*	Model 3^&^
				HR	HR	HR	HR
				(95% CI)	(95% CI)	(95% CI)	(95% CI)
28 days, death	39/414	9/64	1.52 (0.73–3.14)	1.11 (0.53–2.30)	1.05 (0.51–2.18)	1.12 (0.52–2.42)
	Ref					
28 days, discharge	345/414	51/64	0.85 (0.63–1.15)	0.94 (0.70–1.27)	0.96 (0.71–1.30)	1.02 (0.75–1.37)
	Ref					

COPD, chronic obstructive pulmonary disease; DM, diabetes mellitus; CAD, coronary artery disease; SOFA, Sequential Organ Failure Assessment; GCS, Glasgow Coma Scale; HR, hazard ratio.

^#^Model 1 was adjusted for age and sex.

^*^Model 2 was model 1 + chronic conditions of COPD, DM, CAD, hypertension, and moderate to severe kidney disease.

^&^Model 3 was model 2 + home medication of corticosteroids, with symptoms of fever (>37.5°C/99.5°F), receiving corticosteroids between randomization and hospital discharge, receiving tocilizumab between randomization and hospital discharge, receiving azithromycin between randomization and day 8, and standardized total SOFA with GCS at baseline.

**Figure 2:**
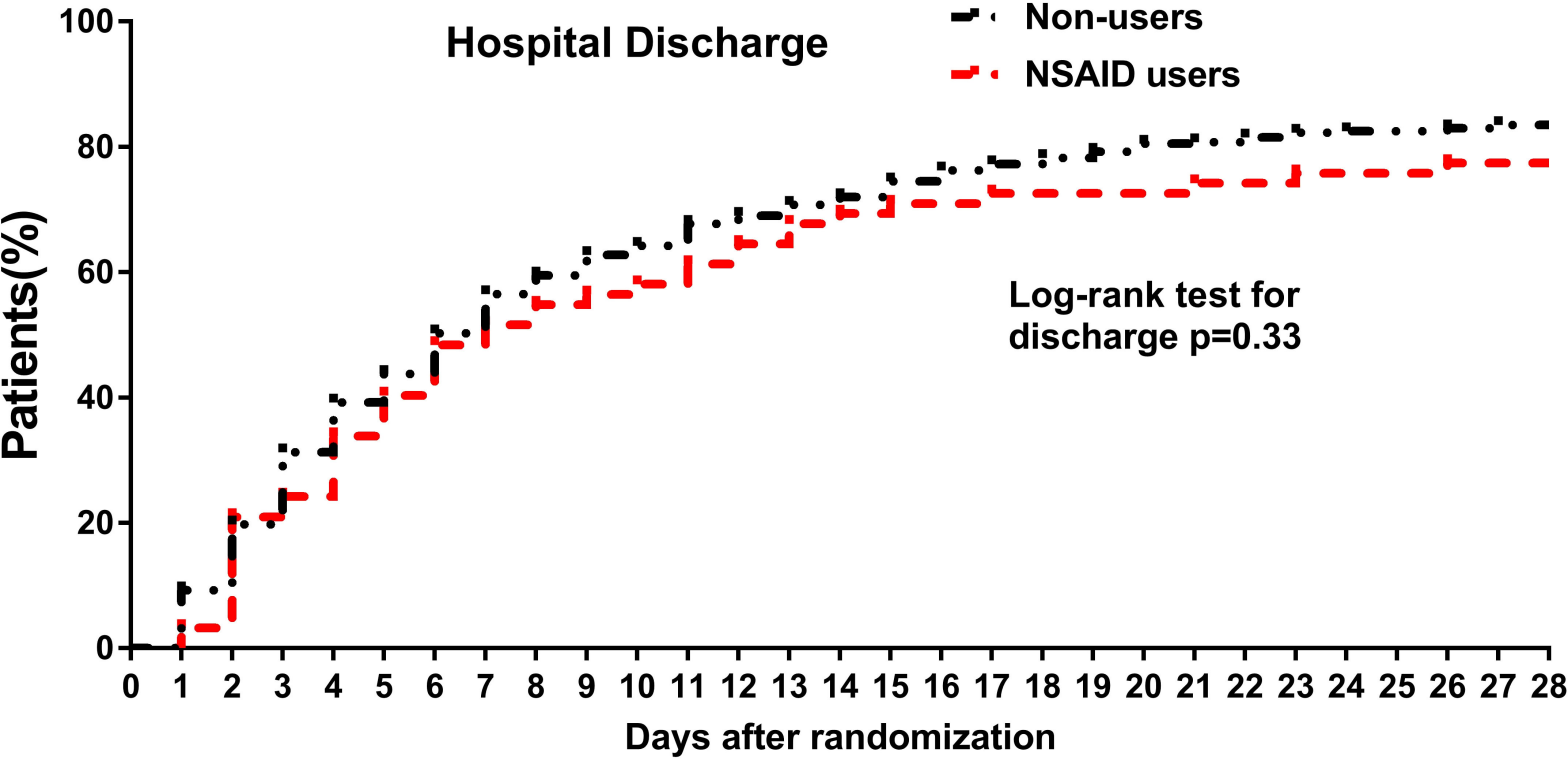
Cumulative incidence curves of hospital discharge through 28 days among NSAID users and non-users among hospitalized patients with COVID-19. For hospital discharge, all patients were followed up to discharge or 28 days after randomization in ORCHID. A patient was considered discharged from the hospital once discharged from the index hospitalization; rehospitalizations were not considered in this analysis. COVID-19, coronavirus disease 2019; ORCHID, Outcomes Related to COVID-19 Treated With Hydroxychloroquine Among Inpatients With Symptomatic Disease; NSAID, non-steroidal anti-inflammatory drug.

The cumulative incidence of hospital discharge through 28 days was 345 for 51. The cumulative hospital discharge curves over 28 days showed no significant difference between NSAID users and non-users (**Figure 2**). Consistently, the time to discharge was not significantly increased among the non-users in the multivariable analysis in all the adjusted models (fully adjusted: *HR*: 1.02, 95% *CI*: 0.75–1.37).

### Safety outcomes with non-steroidal anti-inflammatory drug use in patients with COVID-19

A national cohort found that NSAIDs were associated with an increased risk of stroke in patients with COVID-19 (Jeong et al., 2021). However, in this study, we found no significant association between NSAIDs and stroke (crude *OR*: 0.91, *p* = 0.95). Similarly, no significant association of deep venous thrombosis (crude *OR*: 1.48, *p* = 0.53) and bleeding (crude *OR*: 2.13, *p* = 0.64) with NSAIDs was found.

### Supplemental analysis

As shown in **Supplementary Table S1**, the results were similar when changing 28-day mortality to in-hospital mortality (fully adjusted: *HR*: 0.96, 95% *CI*: 0.45–2.04). Furthermore, there was no significant association between NSAID use and ICU admission (fully adjusted: *HR*: 0.68, 95% *CI*: 0.32–1.41) when excluding patients with ICU admission at baseline.

### Meta-analysis

**Figure 3:**
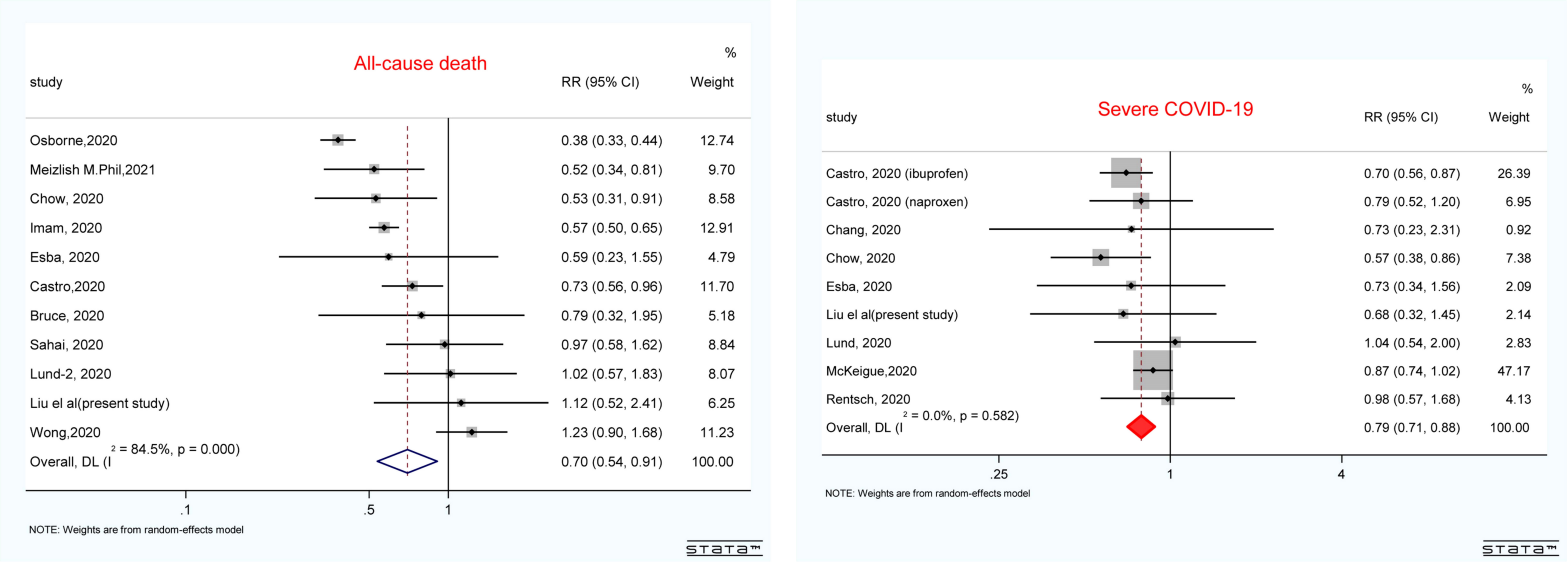
Forest plot showing the pooled results of NSAID use and all-cause mortality and disease severity in patients with COVID-19. The results were pooled by using the random effect. RRs greater than 1.0 indicated less favorable outcomes for patients who are NSAID users compared with non-users. COVID-19, coronavirus disease 2019; RR, risk ratio; NSAID, non-steroidal anti-inflammatory drug.

We included 14 studies involving 48,788 COVID-19 patients with acceptable quality (**Supplementary Figure S1** and **Supplementary Tables S2**, **S3** **)**. The majority of the studies (n = 13) reported the outcomes in the multivariable analysis. As shown in **Figure 3**, non-users were not significantly associated with an increased risk of death (pooled risk ratio (*RR*): 0.70, 95% *CI*: 0.54–0.91) or disease severity (pooled *RR*: 0.79 95% *CI*: 0.71–0.88) among patients with COVID-19. The results were stable by excluding the study of univariate analysis of Subudhi et al. (data not shown). The funnel plot in **Supplementary Figures S2, S3** suggests no significant publication bias.

## Discussion

In this *post-hoc* analysis of multicenter RCTs conducted across 34 hospitals in the United States, we found that NSAIDs are not associated with worse clinical status, including 28-day all-cause mortality and hospital discharge. The conclusion was stable in the supplemental analysis. Furthermore, combined with our results, the meta-analysis also showed that the use of NSAIDs does not have harmful effects on patients with COVID-19, providing both a survival benefit and a severity benefit.

The use of NSAIDs in COVID-19 continues to be debated. The initial concerns originated from a letter that suggested that ibuprofen could exacerbate the prognosis of COVID-19 through the upregulation of ACE2 receptors (Fang et al., 2020). Further fueling this concern, a case report described four young people with COVID-19 who deteriorated after taking ibuprofen in a French hospital (Day, 2020). To date, there is still no consensus on the use of NSAIDs across national health agencies. Our study did not find an increased risk of 28-day mortality, hospital discharge, or ICU admission in hospitalized patients with COVID-19. These results contrast those of a national population-based survey in South Korea (Jeong et al., 2021) but are consistent with those of a national Danish study (Lund et al., 2020) and several observational studies (Lund et al., 2020; Rinott et al., 2020; Abu Esba et al., 2021; Wong et al., 2021). Jeong et al. (Jeong et al., 2021) found that NSAID users, compared with non-users, had a 65% increased risk of the primary composite outcome of in-hospital death, ICU admission, mechanical ventilation use, or sepsis using South Korea’s nationwide healthcare database. However, their national survey might have a significant bias. NSAID users might have more severe symptoms than non-users since the results from their subgroup analysis (a particularly oppositive trend across age subgroups) and sensitivity analysis (a harmful effect was not found compared with individuals administered with paracetamol, a drug used for similar indications as NSAIDs) were not stable. Compared with previous reports, our study was designed using a more homogeneous patient cohort from the ORCHID and had a prospective design. Moreover, the in-hospital medication was adjusted, which also vastly reduced the potential confounding effect, making our results more reliable.

In general, NSAIDs might be a relative contraindication in elderly patients, which results in a lower rate of preadmission NSAID use in older age groups among patients with COVID-19. A recent survey confirmed this phenomenon. However, in our study, NSAID users were older than controls. This inconsistency might come from defining NSAID users as patients with regular home NSAID use, which led us to include more patients taking aspirin in the NSAID group than in the secondary prevention group. This means that the NSAID users in our cohort might have had a higher cardiovascular burden. Although confounding factors, such as cardiovascular diseases, were adjusted for, potential unmeasured confounding factors might have led to an overestimation of the harmful effects of NSAIDs on COVID-19. Thus, our results regarding NSAIDs are conservative. Furthermore, as mentioned above, the definition of our exposure might result in the inclusion of more aspirin users. Due to data limitations, we could not categorize the NSAIDs. However, several studies have shown that several commonly used NSAIDs, including ibuprofen, naproxen, and aspirin, do not have a harmful effect on COVID-19 (Abu Esba et al., 2021; Castro et al., 2020; Chow et al., 2021; McKeigue et al., 2021; Osborne et al., 2021; Wong et al., 2021). The potential mechanism might lie in their ability to inhibit the replication of the virus and their anti-inflammatory properties (Robb et al., 2020; Chen et al., 2021).

Regarding the safety outcomes, we did not find a significant difference in the incidence of stroke. Two observational studies also assessed the use of NSAIDs and stroke in patients with COVID-19, with inconsistent results. Jeong et al. (Jeong et al., 2021) found an increased risk of ischemic stroke with NSAID use in a nationally matched cohort; however, this increase was not significant in another cohort using aspirin (Sahai et al., 2020). Interestingly, among patients with acute respiratory infections, an increased risk of ischemic stroke (*OR*: 2.27) was also found among NSAID users compared with non-users in a case-crossover study (Wen et al., 2018). Therefore, the risk of association between stroke and the use of NSAIDs remains unclear. Notably, the incidence of stroke in patients hospitalized with COVID-19 was low in a recent international retrospective study, and it was shown that ischemic stroke did not increase the risk of death (Nogueira et al., 2021). Therefore, considering that the available cases were limited, the potential association between the use of NSAIDs and stroke and the risk–benefit ratio should be further evaluated in ongoing RCTs or more extensive observational studies. It is also well known that NSAIDs increase the risk of major bleeding (e.g., upper gastrointestinal complications) (Coxib et al., 2013). We did not find an increased risk of bleeding in the present study. These results are consistent with another retrospective cohort study among hospitalized patients with COVID-19, which showed that aspirin was not linked to an increased risk of major bleeding (Chow et al., 2021) in COVID-19. The potential reasons might be explained by the fact that patients with COVID-19 are frequently hypercoagulable, and thrombocytopenia is uncommon in COVID-19 patients. However, considering the limited evidence, the risk of bleeding should be further assessed in ongoing trials (NCT0438276840, NCT0433462941, and NCT04344457).

### Strengths and limitations

The greatest strength of this study was that it was based on a large RCT, with a homogeneous population, complete variance (e.g., in-hospital medication and symptoms of hospital admission), reliable outcome events, and no recall bias. Furthermore, we also conducted a meta-analysis with consistent results that confirmed the lack of potential harmful effects of NSAIDs on COVID-19.

We recognize some possible limitations. First, there are intrinsic limitations associated with any observational study, which cannot prove a causal relationship. Second, NSAID users were defined as those regularly taking NSAIDs as home medication; however, types, duration, dose, and the disease for NSAID prescription were not available, limiting the interpretation of our results. However, the stratified analysis of meta-analysis by the type of NSAID showed that there is no significant difference in the association between NSAID exposure and mortality in patients with COVID-19 (data not shown). Third, the index period of prior hospitalization for COVID-19 was missing; thus, our study might not be generalized to the in-hospital treatment of COVID-19.

## Conclusions

The use of NSAIDs is not associated with worse clinical outcomes, including 28-day mortality, hospital discharge, and ICU admission, in American adult hospitalized patients with COVID-19. The meta-analysis showed a similar conclusion based on current evidence. Based on current evidence, the use of NSAIDs is safe and should not be cautioned against during the COVID-19 pandemic. Ongoing trials should further assess in-hospital treatment with NSAIDs for patients with COVID-19.

## Data availability statement

The original contributions presented in the study are included in the article/**Supplementary Material**. Further inquiries can be directed to the corresponding authors.

## Ethics statement

The study involving participants were reviewed and approved by ORCHID trial. All the patients signed their written informed consent to participate in this study.

## Author contributions

XL and PY were responsible for the entire project and revised the draft. JZ, HS, XT, PX, and ZL performed the data extraction and statistical analysis, interpreted the data, and drafted the first version of the manuscript. MX, JM, and YS revised the manuscript. All authors participated in the interpretation of the results, prepared the final version of the manuscript, and approved the final version to be published. All authors agreed to be accountable for all aspects of the work in ensuring that questions related to the accuracy or integrity of any part of the work are appropriately investigated and resolved.

## Funding

This work was supported in part by the Natural Science Foundation in Jiangxi Province grant (No. 202002BAB216022 to JZ, No. 20192ACBL21037 and No.202004BCJL23049 to PY). This work was supported in part by the National Natural Science Foundation of China (No. 82160371 to JZ and No. 82100869 to PY). All funding agencies had no role in the design, methods, subject recruitment, data collection, analysis, and preparation of the paper.

## Acknowledgments

We thank Outcomes Related to COVID-19 Treated With Hydroxychloroquine Among Inpatients With Symptomatic Disease (ORCHID) investigators for conducting this trial and making these data available. We acknowledge all healthcare workers worldwide involved in the fight against COVID-19.

## Conflict of interest

The authors declare that the research was conducted in the absence of any commercial or financial relationships that could be construed as a potential conflict of interest.

## Publisher’s note

All claims expressed in this article are solely those of the authors and do not necessarily represent those of their affiliated organizations, or those of the publisher, the editors and the reviewers. Any product that may be evaluated in this article, or claim that may be made by its manufacturer, is not guaranteed or endorsed by the publisher.
