# Supplementary material for: Non-steroidal anti-inflammatory drugs and clinical outcomes in patients with COVID-19
Source: Front Cell Infect Microbiol. 2022 Oct 17;12:935280. doi: 10.3389/fcimb.2022.935280 (PMC9618688; doi:10.3389/fcimb.2022.935280)
Supplement: Supplementary file 1 [file DataSheet_1.docx]

**Supplemental Table S1. Supplemental analysis for the association between use of NSADIs and in-hospital mortality, and ICU admission in patients with COVID-19**

| **Outcomes** | **Non-users**  **Cases/N** | **NSAIDs users**  **Cases/N** | **Crude**  **HR** | **Model 1^#^**  **HR** | **Model 2***  **HR** | **Model 3^&^**  **HR** |
| --- | --- | --- | --- | --- | --- | --- |
|  |  |  | **(95% CI)** | **(95% CI)** | **(95% CI)** | **(95% CI)** |
| **In hospital death** | 43/414  Ref | 9/64 | 1.38(0.67-2.38) | 1.03(0.50-2.13) | 1.05(0.51-2.18) | 0.96(0.45-2.04) |
| **ICU admission** | 66/344  Ref | 10/53 | 0.94(0.48-1.84) | 0.91(0.46-1.79)) | 0.96(0.49-1.90) | 0.68(0.32-1.41) |

# Model 1 was adjusted for age and sex

* Model 2 was model1+ chronic conditions of COPD, DM, CAD, hypertension, Moderate to severe kidney disease

&Model 3 was model2+home medication of corticosteroids, with symptoms of fever (> 37.5° C / 99.5° F, receiving corticosteroids between randomization and hospital discharge, receiving tocilizumab between randomization and hospital discharge, receiving azithromycin between randomization and day 8, Standardized Total SOFA with GCS at baseline

**Abbreviations**: COPD, chronic obstructive pulmonary disease; DM, Diabetes mellitus; CAD, Coronary artery disease; SOFA, Sequential Organ Failure Assessment; GCS, Glasgow Coma Scale. HR, hazard ratio; ICU: intensive care unit

**Supplemental Table S2. Included studies of the meta-analysis reporting the use of NSAIDs in patients with COVID-19**

| 1.Abu Esba LC, et al. Ibuprofen and NSAID Use in COVID-19 Infected Patients Is Not Associated with Worse Outcomes: A Prospective Cohort Study. Infect Dis Ther. 2021;10(1):253-68. doi:10.1007/s40121-020-00363-w.  2. Castro, V. M., et al. Brief Report: Identifying common pharmacotherapies associated with reduced COVID-19 morbidity using electronic health records. medRxiv, 2020.2004.2011.20061994, doi:10.1101/2020.04.11.20061994 (2020).  3. Chang, T. S. et al. Prior diagnoses and medications as risk factors for COVID-19 in a Los Angeles Health System. medRxiv, doi:10.1101/2020.07.03.20145581 (2020).  4. Meizlish ML, Goshua G, Liu Y, et al. Intermediate-dose anticoagulation, aspirin, and in-hospital mortality in COVID-19: a propensity score-matched analysis. medRxiv. 2021. doi:10.1101/2021.01.12.21249577  5.Rentsch, C. T. et al. Early initiation of prophylactic anticoagulation for prevention of COVID-19 mortality: a nationwide cohort study of hospitalized patients in the United States. medRxiv, doi:10.1101/2020.12.09.20246579 (2020).  6.Sahai, A. et al. SARS-CoV-2 Receptors are Expressed on Human Platelets and the Effect of Aspirin on Clinical Outcomes in COVID-19 Patients. Res Sq, doi:10.21203/rs.3.rs-119031/v1 (2020).  7.Subudhi, S. et al. Comparing Machine Learning Algorithms for Predicting ICU Admission and Mortality in COVID-19. medRxiv (2020). Prevalent use of NSAIDs: 126/1144  8. Imam, Z. et al. Older age and comorbidity are independent mortality predictors in a large cohort of 1305 COVID-19 patients in Michigan, United States. J Intern Med 288, 469-476, doi:10.1111/joim.13119 (2020).  9.Chow, J. H. et al. Aspirin Use Is Associated With Decreased Mechanical Ventilation, Intensive Care Unit Admission, and In-Hospital Mortality in Hospitalized Patients With Coronavirus Disease 2019. Anesth Analg 132, 930-941, doi:10.1213/ANE.0000000000005292 (2021).  10.Bruce E, Barlow-Pay F, Short R, et al. Prior Routine Use of Non-Steroidal Anti-Inflammatory Drugs (NSAIDs) and Important Outcomes in Hospitalised Patients with COVID-19. J Clin Med. 2020;9(8). doi:10.3390/jcm9082586.  11. McKeigue, P. M. et al. Associations of severe COVID-19 with polypharmacy in the REACT-SCOT case-control study. medRxiv (2020).  12. Lund LC, Kristensen KB, Reilev M, et al. Adverse outcomes and mortality in users of non-steroidal anti-inflammatory drugs who tested positive for SARS-CoV-2: A Danish nationwide cohort study. PLoS medicine 2020; 17:e1003308.  13. Wong AY, MacKenna B, Morton CE, et al. Use of non-steroidal anti-inflammatory drugs and risk of death from COVID-19: an OpenSAFELY cohort analysis based on two cohorts. Ann Rheum Dis 2021.  14. Osborne TF, Veigulis ZP, Arreola DM, Mahajan SM, Röösli E, Curtin CM. Association of mortality and aspirin prescription for COVID-19 patients at the Veterans Health Administration. PloS one 2021; 16:e0246825. |
| --- |

**Supplemental Table S3: Newcastle-Ottawa Scale (NOS) scores for included studies**

| Author, years | selection | | | | Comparability# | Outcome | | | Total |
| --- | --- | --- | --- | --- | --- | --- | --- | --- | --- |
|  | Exposed cohort | None exposed cohort | Ascertainment of exposure | Outcome of interest |  | Assessment of outcome | Length of follow-up | Adequacy of follow-up |  |
| Rentsch, 2020, USA | * | * | * | * | ** | * | * |  | 8* |
| Castro,2020, USA | * | * | * | * | ** | * |  |  | 7* |
| Abu Esba, 2020, Riyadh | * | * | * | * | ** | * | * |  | 8* |
| Wong et al,2020, USA | * | * | * | * | ** | * | * |  | 8* |
| Chow, 2020, USA | * | * | * | * | ** | * |  |  | 7* |
| Osborne,2020, USA | * | * | * | * | ** | * | * |  | 8* |
| Bruce, 2020, UK | * | * | * | * | ** | * | * |  | 8* |
| Meizlish,2020, USA | * | * | * | * | ** | * | * |  | 8* |
| Sahai, 2020, Cleveland | * | * | * | * | * | * |  |  | 6* |
| Subudhi, 2020, USA | * | * | * | * | * | * |  |  | 6* |
| Lund, 2020, Denmark | * | * | * | * | ** | * | * |  | 8* |
| Imam, 2020, USA | * | * | * | * | ** | * | * |  | 8* |
|  | Selection | | | | Comparability | Exposure | | |  |
|  | Case definition | Representative cases | Selection of Controls | Definition of Controls |  | Ascertainment of exposure | Same method | Non-Response rate |  |
| McKeigue,2020, Scotland^*^ | * | * | * | * | ** |  | * |  | 7* |
| Chang, 2020, USA^*^ | * | * |  | * | ** | * | * |  | 7* |

*Case-control study

#Adjusted for age and any other factors


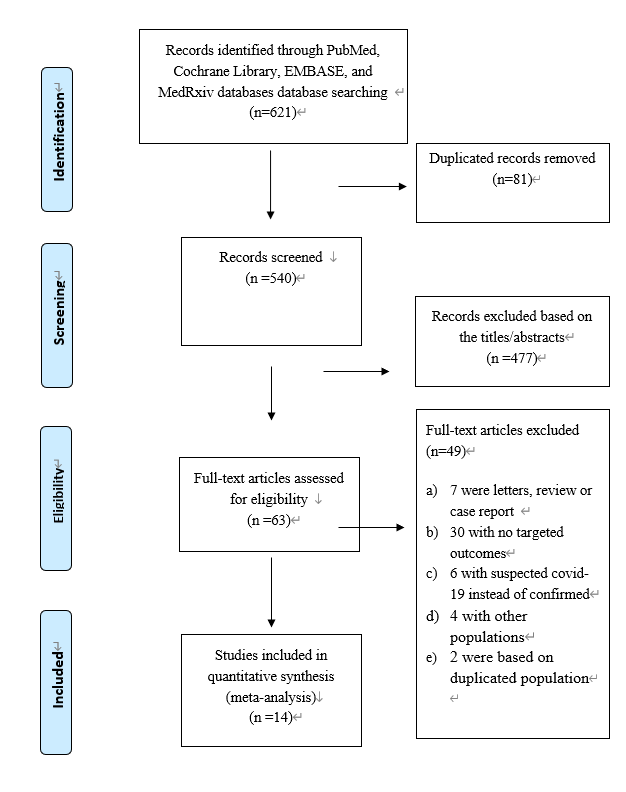


**Figure S1. Flow chart of study selection in the meta-analysis of NSAIDs and clinical outcomes in patients with COVID-19**


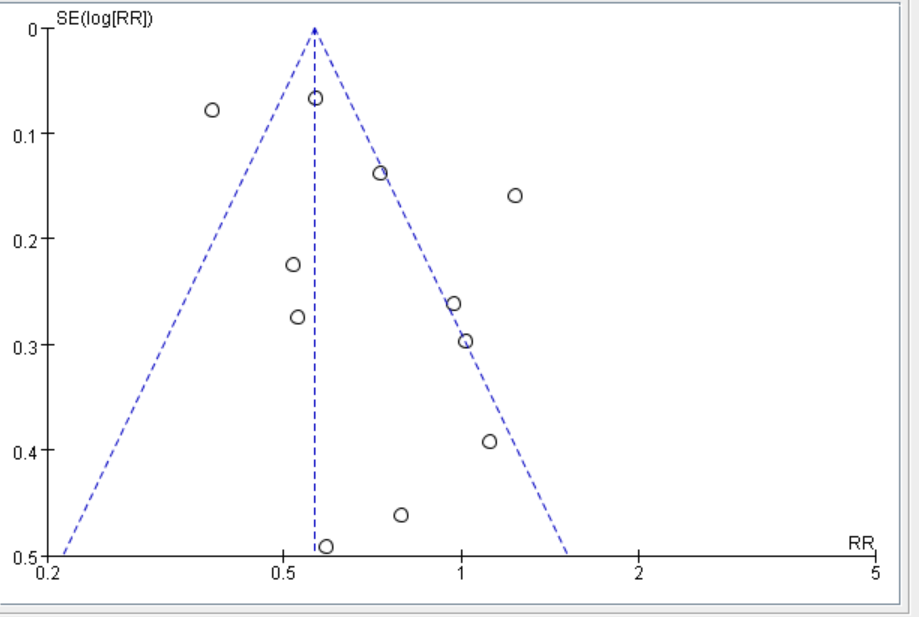


**Figure S2. Publication bias detected by funnel plot for the association of NSAIDs and risk COVID-19 and death in COVID-19 patients.**

NSAID: nonsteroidal anti-inflammatory drugs; COVID-19: Corona Virus Disease 2019;


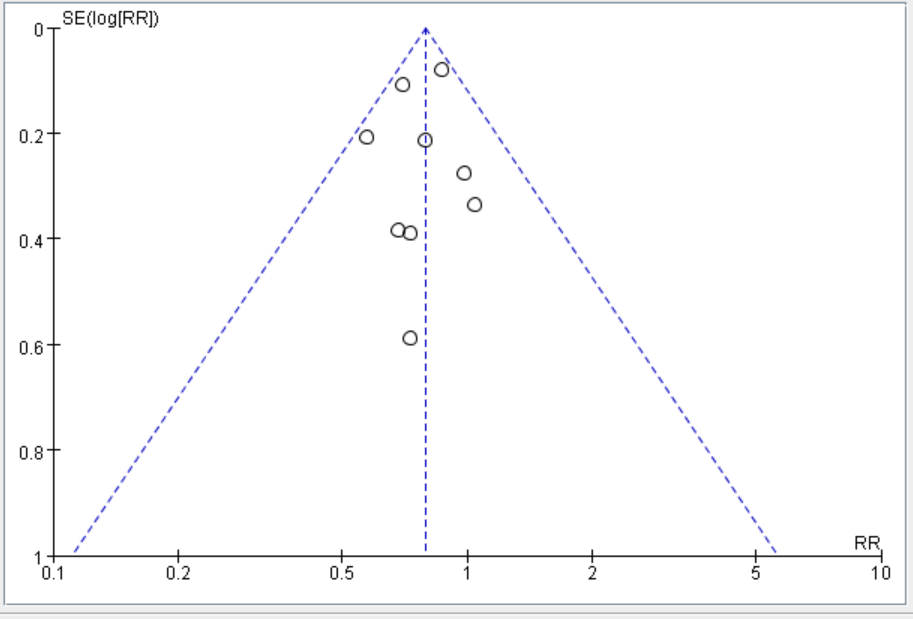


**Figure S3. Publication bias detected by funnel plot for the association of NSAIDs and risk COVID-19 and severity in COVID-19 patients.**

NSAID: nonsteroidal anti-inflammatory drugs; COVID-19: Corona Virus Disease 2019;
